# Editorial: Leishmaniasis: control and elimination, volume II

**DOI:** 10.3389/fcimb.2025.1628558

**Published:** 2025-07-04

**Authors:** Rajiv Kumar, Mary Wilson, Christian Engwerda

**Affiliations:** ^1^ Centre of Experimental Medicine & Surgery, Institute of Medical Sciences, Banaras Hindu University, Varanasi, India; ^2^ Department of Microbiology & Immunology, University of Iowa, Iowa City, IA, United States; ^3^ Immunology & Infection Laboratory, QIMR Berghofer Medical Research Institute, Herston, QLD, Australia

**Keywords:** leishmaniasis, post kala azar dermal leishmaniasis, atypical cutaneous leishmaniasis, immune modulation, mathematical model

Visceral leishmaniasis (VL), caused by *Leishmania donovani*, is a neglected tropical disease that primarily affects impoverished populations living in resource limited regions. Cases in Southeast Asia, India and Nepal contribute significantly to the disease burden. Although the number of new cases has been declining, ongoing efforts are crucial to sustain this. Notably, Bangladesh, once an endemic country, has recently achieved its elimination goals, with no new reported cases in the past three years. A key component of VL elimination strategies has been the identification of potential reservoirs, such as asymptomatic individuals harbouring the parasite and those with post kala azar dermal leishmaniasis (PKDL) or HIV-VL co-infections. However, a growing concern in countries like India, Sri Lanka, Nepal, and Bhutan is the emergence of atypical cutaneous leishmaniasis (ACL) in newly affected regions. Jain et al. illuminate the growing emergence of ACL in traditionally VL endemic regions of Southeast Asia. ACL has been associated with genetic variants of *L. donovani*. Comparative genomic studies have revealed that strains causing ACL are genetically distinct from those responsible for VL. Their findings echo the larger epidemiological warning: *Leishmania* species are adapting, and so must our surveillance systems. Clinically, ACL can be difficult to differentiate from classical cutaneous leishmaniasis (CL), complicating diagnosis. To address these challenges, the implementation of molecular diagnostic techniques at primary health care centres is essential for diagnosis. Furthermore, isolating ACL causing parasites and conducting genome sequencing to compare them with VL causing strains can enhance our understanding of strain diversity. This knowledge may also help address critical concerns, such as the potential conversion of ACL strains into forms capable of causing visceral disease.

The study by Oualha et al. represents a leap forward in computational parasitology. Using machine learning, they screened compounds approved by the FDA and identified several that have high efficacy against *Leishmania* parasites. Among those candidates, some had established safety profiles in humans, highlighting their potential for fast track repurposing. The model employed achieved a high degree of accuracy by integrating structural, pharmacokinetic, and bioactivity features. This study marks a shift from the traditional trial and error drug pipeline to a data driven approach. Such tools may unlock new therapeutic options with low costs more rapidly.

Mathematical modelling and statistical methods have emerged as a tool to predict different epidemiological parameters. The study by Hao et al. marks a significant advancement in how we can evaluate public health interventions. By developing and applying an improved harmonic Poisson segmented regression model, the authors address a long standing challenge of distinguishing genuine intervention effects from seasonal fluctuations in disease incidence. Their model offers representation of real world transmission patterns. When applied to data from 2017 to 2021, the model demonstrated that although VL incidence was rising prior to intervention, the growth rate significantly slowed following the implementation of screening, vector control, and canine management strategies. This work offers evidence that targeted interventions are able to reverse VL case trends. As climate and environmental changes can alter the dynamics of vector borne diseases, tools like this improved regression model will be essential for timely and reliable impact assessment.

In a disease that was reported years ago to be exemplary of Th1/Th2 paradigms, Na and Engwerda’s work offers an interesting immunological update. They reviewed emerging roles for NKG7, a cytolytic granule protein, and TGF-β (transforming growth factor-β), a key immunoregulatory cytokine, in modulating CD4^+^ T cell responses during VL. NKG7 expression was found to be associated with enhanced cytotoxic activity of CD4^+^ T cells, suggesting new roles for T cell mediated parasite killing. Conversely, dysregulated TGF-β signalling may contribute to T cell exhaustion and immune evasion by the parasites. TGF-β is a multifunctional cytokine influencing both regulatory and effector CD4^+^ T cell responses, yet its precise role in VL remains poorly defined. Emerging evidence suggests it may suppress protective immunity by antagonizing NKG7 expression in CD4^+^ T cells. However, the mechanisms and implications of this interaction are still unclear, underscoring the need for focused functional studies. This work highlights a growing appreciation for functional heterogeneity within T cell subsets and offers novel targets for immunotherapeutic intervention. As vaccine efforts continue to lag, insights into immune modulation may prove essential for both prophylaxis and therapy.

Exosomes are tiny vesicles secreted by cells and are emerging as powerful mediators of host pathogen interactions. In this proteomic study, da Silva Lira Filho et al. catalogued novel protein markers in exosomes derived from *Leishmania* spp. Using mass spectrometry and bioinformatics, they identified several candidates with potential diagnostic and immunomodulatory relevance.

What makes this study notable is its translational potential. Exosomal markers could serve as biomarkers for early diagnosis, indicators of treatment response, or even components of subunit vaccines. Moreover, the identified proteins in these exosomes may help elucidate mechanisms by which *Leishmania* manipulates host immunity to promote survival. In the broader context of host–parasite dynamics, this study exemplifies how molecular snapshots can inform both diagnostics and therapeutics in parasitic diseases.

